# Determinants of neural tube defects among women who gave birth in hospitals in Eastern Ethiopia: evidence from a matched case control study

**DOI:** 10.1186/s12905-023-02796-0

**Published:** 2023-12-09

**Authors:** Anteneh Berhane, Tefera Belachew

**Affiliations:** 1 Department of Public Health, College of Medicine and Health Science, Dire Dawa University, Dire Dawa, Ethiopia; 2 Department of Nutrition and Dietetics, Faculty of Public Health, Institute of Health, Jimma University, Jimma, Ethiopia

**Keywords:** NTDs, Preconception folic acid, Predictive factors, NTDs, Adjusted odds ratio

## Abstract

**Introduction:**

Neural tube defects (NTDs) are severe birth defects caused by nutritional, genetic or environmental factors. Because NTDs continue to have a significant health and economic impact on children and community at large, it is crucial to investigate potential risk factors in order to develop novel approaches to NTDs prevention. Determinants for the development of NTDs differ by country, region as well as within the country. The objective of this study was to identify the determinants of NTDs among newborns delivered in three hospitals found in eastern Ethiopia.

**Methods:**

A hospital-based matched case-control study was conducted among 138 cases and 138 control women who delivered in three teaching hospitals in Eastern Ethiopia in 2021. Data were collected using a structured and pre-tested interviewer-administered questionnaire. Cases were mothers who delivered a neonate with any type of NTDs regardless of gestational age or fetal viability, whereas controls were mothers who delivered an apparently healthy newborn. Chi-square was used to assess the significant difference between the two groups. Conditional logistic regression model was used to generate adjusted odds ratio with its corresponding 95% confidence intervals and compare the two groups.

**Results:**

Anencephaly (51.4%) and spinal bifida (34.1%) were the most frequently observed NTDs. None of study participants took preconception folic acid supplementation. Being a non-formal mothers (AOR = 0.34, 95% CI: 0.12–0.92, P = 0.034), rural residence, (AOR = 3.4, 95% CI: 1.18–9.78, P = 0.023), history of spontaneous abortion (AOR = 2.95, 95% CI: 1.15–7.55, P = 0.023), having severe anemia (AOR = 3.4, 95% CI: 1.17–9.87, P = 0.024), history of fever or cold (AOR = 2.75; 95% CI: 1.05–7.15, P = 0.038), and an exposure to various agro-chemicals (AOR = 3.39, 95% CI: 1.11–10.3, P = 0.032) were independent determinants of NTDs.

**Conclusion and Recommendation:**

In this study, NTDs were associated to several determinant factors in the area, including residential area, history of spontaneous abortion, severe anemia, fever/cold, antibiotic use before or during early pregnancy, and exposure to agrochemicals. Addressing the identified determinants is critical in averting the incidence of NTDs in the study area. Moreover, more research is needed to investigate women’s dietary practices as well as the practice of preconception folic acid supplementation for pregnant women in Ethiopia’s current health care system.

## Introduction

Neural tube defects (NTDs) are congenital malformations that occur in the development of the central nervous system during embryonic period [1]. Neural tube formation is central issue to developmental biology with the closure being dependent on the methionine cycle and folate cycle [2]. NTDs can occur in the cranial region (anencephaly, encephalocele), spinal region (spina bifida), or in combination (craniorachisis or complex phenotypes) [3, 4]. NTDs are among the second and most serious congenital anomalies that occur because of incomplete closure of the brain or spinal cord between 12 and 28 days of pregnancy. They are associated with a high rate of neonatal death, morbidity, psychological, emotional, economical problem as well as lifelong disability in survivors and their families [5, 6].

A recent meta-analysis estimated that 260,100 NTDs affected birth outcomes worldwide [7]. Each year, it is estimated that nearly 200,000 neonates are born with NTDs in low and middle-income countries (LMICs) [8]. Another recent meta-analysis research found that, the pooled birth prevalence of NTDs in eastern Africa was 33 per 10,000 births, with Ethiopia having the highest (60 per 10,000 births) and Malawi having the lowest (5 per 10,000 births) [9]. In Ethiopia, the Tigray region had the highest incidence rate of NTDs, accounting for 131 per 10,000 [10] and the eastern part of Ethiopia has the second highest incidence rate (107.5 per 10,000), with the trend increasing alarmingly in recent years [11]. Any woman of childbearing age is at risk of having an NTD-affected pregnancy and it is impossible to determine which women will have NTD-affected pregnancy [12]. Even though the etiology of most NTDs remains undetermined, land mark large -scale randomized randomized clinical trials proofed that genetic, environmental and nutritional risk factors are considered to contribute to their development [13, 14], and among these dietary risk factors play a major role [15]. Mishra, P.R. et al., (2020) reported that 70–95% NTDs are linked to genetics and maternal vitamin intake (MVI) [14]. Over the past 35 years researchers have identified that deficiency of folates at the cellular level may be responsible for NTDs due to disturbed bioavailability of folates, and other nutritional factors, such as trace elements [16]. An increasing body of evidences showed that women who had a previous history of abortions are more likely to develop NTDs [17–21], maternal diabetes [22], and maternal “flu” in the first trimester [23, 24], certain parental occupations [25, 26], are also risk factors of developing NTDs. Omer et al., 2016, observed that the intake of folic acid by the mothers usually starts after conception due to a lack of awareness of its importance is a risk factors for the development of NTDs [27]. Another risk factors for neural tube defects are maternal exposure to valproic acid [28–30]. Maternal hyperthermia in early pregnancy following episodes of maternal fever or heat exposure is also a risk factor for NTDs [31–36]. Different experimental studies showed that hyperglycemia lies within the pathogenic pathway of NTDs, and increasing dietary quality reduced risks of NTDs [37–43]. Exposures to organic solvents; agricultural chemicals, including pesticides; water nitrates; heavy metals such as mercury; ionizing radiation; and water disinfection by-products [44–51] are independent factors for incidence of NTDs. Recently, there is an increase in understanding of risk factors for development of NTDs, and preventative and treatment approaches have witnessed great advances throughout the years. Even though, these risk factors have been identified in Ethiopia, there are not well established the risk factors of NTDs in the real context of the whole country including eastern Ethiopia, which has the second highest burden with an increasing trend in a recent year. Thus, the current study aimed to identify the determinants of NTDs among women who delivered in three hospitals in eastern Ethiopia.

## Materials and methods

### Study setting

The study was conducted in Dilchora Referral Hospital, Hiwot Fana Specialized Teaching Hospital, and Adama Hospital Medical College, found in the eastern part of Ethiopia. Dilchora Referral Hospital is found in Dire Dawa city administration, which is 515 km away from Addis Ababa, the capital of Ethiopia, and serves approximately five million populations from neighboring regions, including Oromia and Somali regions. Hiwot Fana Specialized Teaching Hospital is found in the Harari Region, which is 526 km away from Addis Ababa, and delivers services to the entire community of eastern Ethiopia. In addition, the hospitals also serve as teaching centers for health and medical science students. Adama Hospital Medical College is found in the Oromia Region. It serves as a referral center for more than 6 million people from different regions neighboring zones and regions, including Afar, Amhara, and Somali.

### Study design

A matched case-control design was used to address the objective of this study. Cases and controls were enrolled in the obstetrics/gynecology ward and Neonate Infant and Child Unit (NICU) from March to October 2021.

### Study participants

All newborns in the selected hospitals who fulfilled the case, and control criteria were included. Newborns whose mothers were very sick, emotionally upset during data collection, or died after delivery were excluded from this study.

### Sample size determination and procedure

The sample size was calculated assuming an equal number of cases and controls (1:1), odds ratio of 3.0, power of 80%, 95% confidence level of and non-response rate of 5%. The final sample size was 276 (138 cases and 138 controls). Cases were ascertained prospectively until the calculated sample size was reached. Control neonates were randomly selected from the same hospital. Cases and apparently healthy control neonates (1:1) were matched for a neonate’s sex, and maternal age.

### Operational definition

#### Neural tube defects (NTDs)

is defined as any newborn baby or terminated with anencephaly or spinal bifida, or encephalocele, or meningocele or mylemeningocele.

#### Cases

Mothers, who gave birth to a neonate with any type of NTDs, irrespective of gestational age and fetal survivorship at birth.

#### Control

Mothers who gave birth to a neonate without NTDs who are apparently healthy.

### Data collection procedure and tool

The data from the cases were collected after the mothers delivered a neonate or had terminated her pregnancy due to NTDs in the labor, gynecology ward, and NICU. Control mothers were interviewed randomly within 48 hrs of birth without discrimination regarding their ethnicity, religion, or marital status. A pretested structured questionnaire prepared in the local language and translated to English by an independent translator to check its consistency. Eight trained midwives who worked in the gynecology, obstetrics ward and neonate,infant, and child care unit (NICU) were asked participated mothers about their socio-demographic and medical history data, including, reproductive history, ANC follow-up, obstruction, maternal illness, drug history, preconceptional folic acid, environmental factors, and neonatal status.

### Data quality control

The questionnaire was pre-tested before the actual data were collected and the necessary adjustments were made based on the results of the preliminary tests. Before starting the actual data collection, two days extensive training was given for data collectors and supervisors. Data were collected using the KoBo Tool application via mobile device. The data were checked before leaving the data collection site for immediate action.

### Data processing and analyses

Data were cleaned and analyzed using SPSS for Windows 25. Descriptive analysis was presented using means, frequencies and percentages. To assess the significant difference between the two groups, Pearson and Fisher exact chi-square test was used for comparing catagorical variables. For continous variables, either a paired t test or Wilcoxon Signed rank test was used. All assumptions were checked. A multivariable conditional logistic regression model was used to identify the independent determinants of NTDs. The measure of association of each variable was determined using a parameter of adjusted odds ratio with 95% confidence intervals. Statistical significance declared at P-value < 0.05. A standard error of > 2.0 was used to test for multicollinearity. Model’s fitness was assessed using the Hosmer and Lemeshow tests with P > 0.05 used as fit.

## Results

Of 138 cases, 30(21.7%), 22(15.9%), and 86(62.3%) were from Dil Chora teaching hospital, Hiwot Fana specialization teaching hospital, and Adama medical college hospital, respectively. The overall Mean ± (S.D.) age of mothers was 26.2 ± 5.9 years. Rural resident women were found to be more prevalent than urban resident women (53.6% vs. 46.4%) (P = 0.023). Besides, maternal age 18–24 years was lower among cases (26.8%) as compared to controls (41.3%) (*P* = 0.036). Regarding the family size, most of the mothers who were part of households with 1–5 people were higher among cases (79.7%) as compared to controls (64.5%) (P = 0.005) (Table 1).

**Table 1:** Demographic data of the cases and controls in Eastern Ethiopia

Variables	Categories	Cases		Controls		*P*
		n	%	n	%	
Marital Status	Married	134	97.1	135	97.8	0.605 ^a^
	Divorced	3	2.2	3	2.2	
Maternal age	18–24	37	26.8	57	41.3	0.036
	25–34	67	48.6	86	62.3	
	> 34	14	10.1	15	10.9	
Partner age	20–34	110	79.7	111	80.4	0.88 ^a^
	> 35	28	20.3	27	19.6	
Partner educational status	Non formal education	9	6.5	8	5.8	0.54 ^a^
	Formal (1–12)	106	76.8	99	72.3	
	College and above	23	16.7	30	21.9	
Occupational Status of mother	Governmental	13	9.4	9	6.5	0.695 ^a^
	Madam	90	65.2	89	64.5	
	Private	15	10.9	20	14.5	
	Daily worker	20	14.5	20	14.5	
Partner occupational status	Governmental	27	19.6	31	22.5	0.259 ^a^
	Private	34	24.6	44	31.9	
	Daily worker	14	10.1	16	11.6	
	Farmer	63	45.7	47	34.1	
Blood relation with your partner	No	133	96.4	133	96.4	1.000 ^b^
Family size	1–5 > 5	110 28	79.7 20.3	89 49	64.5 35.5	0.005 ^a^

^a^Pearson chi square; ^b^Fisher Exact test. Significant at < 0.05

Among cases, 97 (70.3%) of mothers were hade 1–3 years gap between the previous pregnancy. The proportion of primigravida women in the cases was higher than in the control group (18.1% vs. 8%) and the difference was statistically significant (p = 0.012) (Table 2).

**Table 2:** Obstetric and health characteristics of case and controls in Eastern Ethiopia

Variables	Categories	Cases		Controls		P
		n	%	n	%	
Gap between the previous pregnancy	Nulligravida	30	21.7	12	8.7	0.011 ^a^
	1–3 years	97	70.3	113	81.9	
	4–7 years	11	8	13	9.4	
Gravidity	Primigravida	25	18.1	11	8	0.012 ^a^
	Multigravida	113	81.9	127	92	
Family planning	No	87	63	99	71.7	0.123 ^a^
	Yes	51	37	39	28.3	
Current Pregnancy	Unplanned	120	87	118	85.5	0.727 ^a^
	Planned	18	13	20	14.5	
ANC visit	No	69	50	64	46.4	0.547 ^a^
	Yes	69	50	74	53.6	
IFA supplement	No	73	52.9	67	48.6	0.47 ^a^
	Yes	65	47.1	71	51.4	
Breast feeding above 2 years	No	128	92.8	130	94.2	0.626 ^a^
	Yes	10	7.2	8	5.8	
Male gender predominance	No	126	91.3	122	88.4	0.425 ^a^
	Yes	12	8.7	16	11.6	
History of preterm	No	120	87	121	87.7	0.856 ^a^
	Yes	18	13	17	12.3	
Chronic hypertension	No	138	100	136	98.6	0.498 ^b^
Gastric disease	No	121	87.7	115	83.3	0.305 ^a^
	Yes	17	12.3	23	16.7	
Suffered with stress	No	120	87	115	83.3	0.397 ^a^
	Yes	18	13	23	16.7	
Suffered with viral infection	No	122	88.4	124	89.9	0.69 ^a^
	Yes	16	11.6	14	10.1	
Suffered with malaria	No	132	95.7	136	98.6	0.28 ^a^
	Yes	6	4.3	2	1.4	
Suffered from parasitic infection	No	104	75.4	106	76.8	0.77 ^a^
	Yes	34	24.6	32	23.2	
Passive cigarette smoker or smoker	No	107	77.5	108	78.3	0.88 ^a^
	Yes	31	22.5	30	21.7	
Partner exposure to chemicals	No	127	92	124	89.9	0.52 ^a^
	Yes	11	8	14	10.1	
Heating/ cooling fumes in living quarters	No	122	88.4	121	87.7	0.85 ^a^
	Yes	16	11.6	17	12.3	
Exposure to radiation	No	125	90.6	124	89.9	0.83 ^a^
	Yes	13	9.4	14	10.1	
Inadequate ventilation during heating	No	124	89.9	128	92.8	0.39 ^a^
	Yes	14	10.1	10	7.2	

^a^Pearson chi square; ^b^Fisher Exact test. Significant at < 0.05, IFA = Iron- folic acid, ANC = Antenatal care

### Type of NTDs

**Fig. 1:**
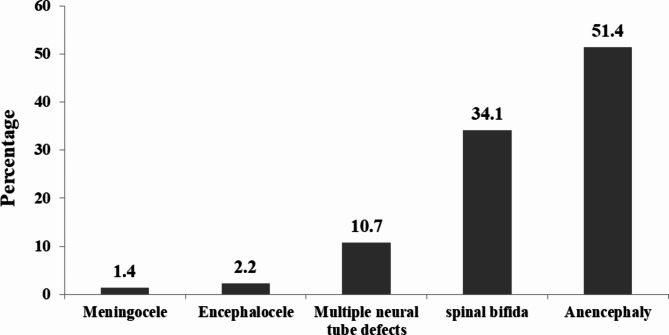
Type of NTDs among deliveries in hospital in Eastern Ethiopia

Of the total NTDs, 51.4% and 34.1% were anencephaly and spinal Bifida, respectively **(**Fig. 1**)**.

### Determinants of NTDs

After controlling for confounding variables, multivariate conditional logistic regression analysis identified that mothers who lived in rural areas, had formal education, had a history of elective or terminated abortions, suffered from severe anemia and fever or cold in pre or early pregnancy, and were exposed to various agro-chemicals were significantly associated with the development of NTDs (Table 3).

**Table 3:** Multivariable analysis for the determinants of NTDs among women who gave birth in hospitals in Eastern Ethiopia

Variables	Cases		controls		*P*	*AOR(95% CI)*
	n(%)		n(%)			
Residence Urban Rural	64(46.4) 74(53.6)		86(62.3) 52(37.7)		*0.023*	1.00 3.41(1.18–9.78)
Educational status of mothers No informal education Formal (1–12) College & above	27(19.6) 100(72.5) 11(8)		26(18.8) 98(71) 14(10.1)		*0.03* *0.272*	1.00 0.34(0.12–0.92) 0.41(0.08–1.99)
History of elective or terminated or spontaneous abortion No Yes	111(80.4) 27(19.6)		122(88.4) 16(11.6)		*0.023*	1.00 2.95(1.15–7.55)
Suffered with Anemia (Before or early pregnancy) No Yes	103(74.6) 35(25.4)		117(84.8) 21(15.2)		*0.024*	1.00 3.4(1.17–9.87)
Fever(hyperthermia)/cold (Before or early pregnancy) No Yes	98(71) 40(29)		107(77.5) 31(22.5)		*0.038*	1.00 2.75(1.05–7.15)
Use any antibiotic (Before or early pregnancy) No Yes	112(81.2) 26(18.8)		122(88.4) 16(11.6)		*0.003*	1.00 6.6(1.89–23.02)
Maternal exposure to agrochemicals No Yes	112(81.2) 26(18.8)		120(87) 18(13)		*0.032*	1.00 3.39(1.11–10.3)

Significant at P < 0.05, AOR = Adjusted odd ratio, Max Std.err = 0.785. CI: Confidence interval

Mothers living in rural areas were 3.4 times higher odds of developing NTDs compared to mothers living in urban areas (AOR = 3.4, 95% CI: 1.18–9.78, P = 0.023). Mothers who had history of elective termination were nearly three times more likely to develop NTDs (AOR = 2.95, 95% CI: 1.15–7.55, P = 0.023) than those who had no history of elective termination.

The risk of having neonates with NTDs was 3.4 higher (AOR = 3.4, 95% CI: 1.17–9.87, P = 0.024) in mothers who suffered from severe anemia in pre or early pregnancy compared with their counterparts. Mothers who had a history of fever in pre or early pregnancy were 2.75 times more likely to have a neonate with NTDs (AOR = 2.75; 95% CI: 1.05–7.15, P = 0.038) than mothers who had no history of fever in pre or early pregnancy.

The risk of having neonates with NTDs was nearly 3.4-fold higher (AOR = 3.39, 95% CI: 1.11–10.3, P = 0.032) in mothers exposed to various agrochemicals compared with their counterparts. The odds of having NTD was 66% lower among mothers who had attended education from grade 1–12 (AOR = 0.34, 95% CI: 0.12–0.92, P = 0.034) compared to illiterate ones.

## Discussion

The objective of this case-control study was to identify the determinants of NTDs. In this study, women who resided in rural areas had higher odds of newborns with NTDs compared to their urban counterparts. This finding is supported by a study conducted in Tigray and Amhara Regional State of Ethiopia [20, 49]. This disparity in residence area could be attributed to differences in education level, health awareness, workload, and stress, as well as economic and cultural factors.

We also found that women with non-formal education had greater odds of newborns with NTDs compared to counterparts. This finding is in line with a study conducted in other studies [20, 52]. Lunau et al., (2015) also reported that there was a significant relationship between women who had lower education and exposed to higher levels of work stress [53]. Experiencing stress before or after early pregnancy could be a predictor for development of NTDs. It was hypothesized that maternal stress could increase the circulating adrenocorticotropin and cortisol levels and affects the fetus’s neural development [54]. This hypothesis was also supported by a systematic review and meta-analysis conducted by Jia et al. (2019) and Suarez and Lucina et al. (2003) [55, 56].

Another potential mechanism associated with the damaging effects of stress may be altered micronutrient concentrations via its influence on nutrient stores in the body [57], including folic acid and other nutrients that are responsible for development of NTDs.

Maternal history of elective or termination or spontaneous abortion was significantly associated with having NTDs affected pregnancy. Our study agreed with other studies [17, 18, 58]. The epidemiologic study showed that a low serum or plasma folate level was associated with an increased risk of early spontaneous abortion [59–61], which could be a level of maternal serum folic acid responsible for spontaneous abortions and development of NTDs. Moreover, maternal serum and erythrocyte folate concentration decreases from the twenty weeks of pregnancy onwards and remain low for a long time after delivery [62–64], which could the reason for developing NTDs in the next pregnancy particularly for women with short inter-pregnancy intervals. Hence, periconceptional folic acid supplements can effectively prevent not only the occurrence of NTDs and but also spontaneous abortion during early pregnancy.

However, Golalipour et al. (2014), De marco et al. (2014) and Todoroff et al., (2000) reported that there was no association between prior spontaneous abortion and development of NTDs [65–67]. This discordant finding between studies could be attributed to the methodological approaches: used.

Mothers with suffered from chronic anemia was also significantly associated with NTDs in this study. The possible mechanism by which chronic anemia before or during early pregnancy could increase NTDs risk in neonate may be through elevated maternal serum homocysteine and the disturbance of methylation process. Disturbance of maternal fetal serum homocysteine and methylation may be responsible for the development of NTDs in the fetus. Various dietary factors, such as folic acid and vitamin B_12_ influence serum homocysteine levels and play a role in the methylation pathway [68–73]. Ferritin can also modulate folate availability via the cellular one-carbon pathway, implying that low iron status can alter folate utilization even when adequate folate intake and extracellular folate concentrations are present [74, 75]. Serum homocysteine levels and methylation seem to be positively correlated with folate deficiency [76]. Iron deficiency is the most common cause of anemia [77–79], which could possibly play a role in development of NTDs in humans. However, the need for more evidence to substantiate this pathway was suggested [80] and showed no significant difference in maternal ferritin or hemoglobin concentrations between NTD-affected and non-affected pregnancies [81]. To know more, further investigation of the mechanism that connects anemia and NTDs will be needed.

We also observed a significant association between fever/cold before or early pregnancy and developing NTDs, which is consistent with the report of studies conducted in various settings around the world [82–84]. A possible physiological mechanism for the association between fever/hyperthermia and the development of NTDs may be that fever is a marker of another underlying process. Specific infections or immune disturbances contribute to increased risk for NTDs. Fever is also associated with increased levels of pro-inflammatory cytokines and other molecules [85] that cross the placenta to affect fetal brain development via mechanisms other than hyperthermia.

This study also found that the use of antibiotics before or early pregnancy was associated with NTDs. The possible reason is that antibiotic medications may have antifolate effects [86]. Some specific forms of antibiotics such as sulfonamides are risk for fetus developing NTDs [87, 88]. However, our finding was not in agreement with a study conducted by Wang M et al., (2014) [89]. Nevertheless, there is no clear mechanism that antibiotics are risk factors for the development of NTDs.

It was also observed that maternal exposure to agrochemicals before or during the early pregnancy period was associated with increased the odds of NTDs in the offspring, which is supported by other studies [17, 50, 90]. The possible explanation could be that agricultural chemicals are lipophilic and alter cell proliferation and differentiation during neurulation [91], averted neurological development and impairment. Agricultural pesticides enter the food chain through animal food sources and crops, including run-off in water bodies [92]. Agricultural chemicals are used more frequently as part of livelihood activities in the study area, implying the need for more research before dismissing this potentially massive exposure. In contrast, a study conducted from a similar case control study in Tigray, Ethiopia, found no significant association between agro-chemicals and NTDs [49]. The strength of this investigation was that both cases and controls were drawn from similar settings in a 1:1 ratio to avoid selection bias. Furthermore, a strong case ascertainment was used to identify NTD cases. An effort was also made to investigate several factors that could be potential determinants, which would contribute to a clear understanding of the risk factors for development of NTDs.

Despite all its significant findings this study has some limitations. The first limitation was the exposure status of study participants was determined, retrospectively, which could be influenced by recall bias. The second limitation of this study is also that biomarkers and genetic polymorphism were not addressed. Moreover, due to the nature of facility-based research, generalization to the general population is difficult. The third limitation was that there was insufficient evidence to support our claim that fever/cold, anemia, and elective termination were associated with the development of NTDs. Moreover, due to the presence of very small numbers in some categories of predictive variables, the model estimates may be unstable which should be interpreted carefully.

## Conclusion

The study found that anencephaly and spinal Bifida are the two most common types of NTDs in the region. The results indicated that the development of NTDs was associated with residence area, history of abortion, history of severe anemia, history of fever, any antibiotics used before or during early pregnancy, and exposure to agrochemicals. Though we did find that dietary factors were a leading causes for developing a NTDs in the study area. As a result, our findings suggest that mulitisectoral efforts should be made to intervene the dietary factors and to control environmental factors such as agrochemicals that contaminate food and water sources. These should be considered as a foundation for public health promotion in the prevention of NTDs. In addition, behavior change interventions based on various strategies, on preconception folic acid or iron-folic acid supplementation should be implemented at the community, school, and health facility levels should be implemented to curb the emerging burden of NTDs in Ethiopia. In general, nutrition intervention including, mandatory iron and folic acid fortification using common vehicles like flour, salt and oil or practicing good dietary iron and folate food sources or preconception iron-folic acid supplementation is required. Furthermore, engage a global network of partners who are experts in conducting NTD prevention programs to track, monitor blood folate concentrations, and develop intervention programs to increase the amount of iron and folic acid consumed by women of reproductive age. Moreover, a multi-sectoral effort aimed at reducing the risk of NTDs is needed in the study area is needed. Dietary practice among cohort women who gave birth with and with out NTDs should be studied in the study area and at the national level.

## Data Availability

All data generated or analyzed during this study are included in this published article.

## Abbreviations

AOR: Adjusted matched odds ratio
ANC: Antenatal Care
CA: Congenital Anomalies
CI: Confidence Interval
FSI: Folate Supplementation Intake
IFA: Iron- Folic Acid
MVI: Maternal Vitamin Intake
NICU: Neonate and Infant care Unit
NTDs: Neural Tube Defects
OR: Odds ratios
SDG: Sustainable Development Goal
